# Oil Palm’s Empty Fruit Bunch as a Sorbent Material in Filter System for Oil-Spill Clean Up

**DOI:** 10.3390/plants11010127

**Published:** 2022-01-04

**Authors:** Nurul Aini Puasa, Siti Aqlima Ahmad, Nur Nadhirah Zakaria, Khalilah Abdul Khalil, Siti Hajar Taufik, Azham Zulkharnain, Alyza Azzura Azmi, Claudio Gomez-Fuentes, Chiew-Yen Wong, Noor Azmi Shaharuddin

**Affiliations:** 1 Department of Biochemistry, Faculty of Biotechnology and Biomolecular Sciences, Universiti Putra Malaysia, Serdang 43400, Malaysia; nurulainipuasa@gmail.com (N.A.P.); aqlima@upm.edu.my (S.A.A.); nadhirahairakaz@gmail.com (N.N.Z.); hajartaufik21@gmail.com (S.H.T.); 2 Laboratory of Bioresource Management, Institute of Tropical Forestry and Forest Products (INTROP), Universiti Putra Malaysia, Serdang 43400, Malaysia; 3 Faculty of Applied Sciences, School of Biology, Universiti Teknologi MARA, Shah Alam 40450, Malaysia; khali552@uitm.edu.my; 4 Department of Bioscience and Engineering, Shibaura Institute of Technology, College of Systems Engineering and Science, 307 Fukasaku, Minumaku, Saitama 337-8570, Japan; azham@shibaura-it.ac.jp; 5 Faculty of Science and Marine Environment, Universiti Malaysia Terengganu, Kuala Nerus 21030, Malaysia; alyza.azzura@umt.edu.my; 6 Department of Chemical Engineering, Universidad de Magallanes, Avda. Bulnes, Punta Arenas 01855, Chile; claudio.gomez@umag.cl; 7 Center for Research and Antarctic Environmental Monitoring (CIMAA), Universidad de Magallanes, Avda. Bulnes, Punta Arenas 01855, Chile; 8 School of Health Sciences, International Medical University, Bukit Jalil, Kuala Lumpur 57000, Malaysia; wongchiewyen@imu.edu.my; 9 Institute of Plantation Studies, Universiti Putra Malaysia (UPM), Serdang 43400, Malaysia

**Keywords:** agriculture waste, diesel spills, sorption capacity, absorbed, fibre, treated

## Abstract

Oil pollution such as diesel poses a significant threat to the environment. Due to this, there is increasing interest in using natural materials mainly from agricultural waste as organic oil spill sorbents. Oil palm’s empty fruit bunch (EFB), a cost-effective material, non-toxic, renewable resource, and abundantly available in Malaysia, contains cellulosic materials that have been proven to show a good result in pollution treatment. This study evaluated the optimum screening part of EFB that efficiently absorbs oil and the physicochemical characterisation of untreated and treated EFB fibre using Fourier Transform Infrared Spectroscopy (FTIR) and Scanning Electron Microscopy (SEM). The treatment conditions were optimised using one-factor-at-a-time (OFAT), which identified optimal treatment conditions of 170 °C, 20 min, 0.1 g/cm^3^, and 10% diesel, resulting in 23 mL of oil absorbed. The predicted model was highly significant in statistical Response Surface Methodology (RSM) and confirmed that all the parameters (temperature, time, packing density, and diesel concentration) significantly influenced the oil absorbed. The predicted values in RSM were 175 °C, 22.5 min, 0.095 g/cm^3^, and 10%, which resulted in 24 mL of oil absorbed. Using the experimental values generated by RSM, 175 °C, 22.5 min, 0.095 g/cm^3^, and 10%, the highest oil absorption achieved was 24.33 mL. This study provides further evidence, as the data suggested that RSM provided a better approach to obtain a high efficiency of oil absorbed.

## 1. Introduction

Oil pollution such as diesel poses a major threat to the marine environment. Many factors contribute to the seawater’s oil pollution, including the transportation of supertankers with diesel fuel storage in bulk quantities, which can significantly increase the risk of pollution from events such as accidental spills and leakages [1]. Recently, a tanker carrying around one million barrels of crude oil was involved in a collision that resulted in an oil spill in the Yellow Sea, China, which could cause major environmental damage [2]. Due to the risk of ecological disaster, oil spills should be prevented as they pose serious health risks to the environment, humans, and animals [3,4,5]. 

The oil contains direct-acting compounds such as mono- and dinitropolyaromatic hydrocarbons that lead to mutagenic effects when exposed [6]. Diesel oil is highly hazardous as it contains carcinogenic substances that include polycyclic aromatic hydrocarbons (PAHs), a highly condensed aromatic hydrocarbon [7]. After a spill, the oil can stay for decades as it decomposes slowly in low-temperature regions, thus becoming more harmful [8,9]. As the hydrocarbon contaminant is highly persistent with some toxicity packages, this can have long-term effects on many cold temperate species and further destroy the ecosystem. Thus, oil spills have created a great need for remediation efforts to be considered [10,11,12].

Several remediation methods can be used to remove oil contaminants from the environment; they include: physical, chemical, and biological methods. The physical and chemical methods are non-favourable methods to remove contaminants as the methods are expensive and result in an incomplete removal [13]. Other than the physical and chemical remediation, bioremediation methods can be used as one of the remediation processes. Bioremediation is a process where the contaminants are biologically removed from the contaminated sites. Opposite to physical and chemical remediation, bioremediation promises more eco-friendly treatment as cost-effective and environmentally sustainable methods [14].

Recently, there has been increasing interest in using cheap, effective, non-toxic, and abundant natural materials, mainly from agricultural waste as organic oil spill sorbents [15]. The potential use of this natural oil clean-up sorption technology gives a significant possibility for high efficiency of oil removal from the sea’s surface with no secondary pollution produced. It is environmentally friendly apart from giving minimum harmful effects to the ecosystem. The use of agricultural waste as a biosorbent is promising due to its efficient disposal and cost-effectiveness [16,17]. The natural sorbent can be biologically degraded, abundantly available, and relatively cheap, thus becoming a significant advantage compared to the synthetic sorbent. The use of agricultural waste as a biosorbent can reduce the waste from the industry. The natural sorbents that contain cellulosic materials such as cotton [18], palm fibres [19], and pineapple leaves [20] have been proven to have a good result in oil spill treatment.

A tree, *Elaeis guineensis*, produces the most commonly used vegetable oil in the world today, palm oil. Indonesia, Malaysia, Thailand, Colombia, and Nigeria are the five top oil palm producers [21]. Malaysia and Indonesia are the biggest producers and exporters of palm oil and palm oil products globally [22]. The solid wastes from the oil palm industries are EFB, mesocarp fruit fibres (MF), and palm kernel shells (PKS) [16]. After being pressed to collect oil, more than 70% of fresh fruit brunch components are left as EFB, making them the most abundant wastes from palm oil industries [23]. The presence of this EFB as oil palm waste in large quantities has created a significant disposal problem that can pollute the environment. 

EFB consists of hard, abundant, and multicellular solid fibres. The rough and jagged morphology of EFB makes it suitable for biosorption, and it has been reported that EFB is widely used to biosorp heavy metals and dyes. Nonetheless, its use in oil biosorption remains limited [24]. EFB is an eco-friendly material and is generally used to produce conventional biocomposite products (moulded product panel, plywood, fibreboard, hybrid biocomposite) and advanced biocomposites (thermoplastics, thermosets, and elastomers) [25]. 

EFB consists of 80% of the stalk (fibre) and 20% of the spikelet. In spikelet and stalk, their components are regarded as lignocellulose biomass composed of lignin, hemicellulose, and cellulose [26]. The chemical compositions of EFB fibre are cellulose (44.2%), hemicellulose (33.5%), and lignin (20.4%) [27]. The properties of EFB include strong structural stability and the ability to adsorb contaminants such as heavy metals and dye [28,29]. Recently, the interest in innovations of chemical, physical, and biological methods for lignocellulosic materials oil palm biomass for value-added products has been growing [25]. EFB is widely used to biosorp heavy metals and dyes, but its use in oil biosorption remains limited. As cellulose is widely used as an adsorbent, research into the use of EFB to biosorp oil as potential uses in oil-spill cleaning is timely. 

This study aimed to evaluate the ability of EFB fibres to clean up oil spills. The experiments were conducted in a hybrid system with various parameters. Sorption capacity, and oil and water absorbed efficiency were investigated from the temperature, time, packing density, and oil concentration parameters. Another aim of this work was to analyse the characterization of EFB samples before and after being treated, with and without oil, using Fourier Transform Infrared Spectroscopy (FTIR) and Scanning Electron Microscope (SEM).

## 2. Materials and Methods

### 2.1. Materials

Empty fruit bunch agricultural waste was collected from the local oil palm industry, Manjung, Perak, Malaysia. The samples were kept at room temperature until further use. Diesel fuel (Dynamic diesel fuel Euro 5) was bought from PETRONAS UPM Serdang, Selangor, Malaysia. The seawater was obtained from Port Klang (2.9999° N, 101.3928° E) with salinity of 15.19 ppt and pH 7.5–8.1.

### 2.2. Laboratory Scale Set Up and Sorbents Selection

A plastic bottle (250 mm × 50 mm) was used as a filter column; 5 g of each sample were placed inside a cylinder spacer (h = 10 cm, d = 5 cm) made from PVC mesh wire and inserted in the column; 40 mL of diesel was vigorously mixed in 400 mL of seawater, poured into the column, and left to stand aside for 10 min after each run. The oil and water effluent was observed and measured together with the weight of bundled samples. The sorption capacity (Equation (1)) and oil and seawater absorbed efficiency (Equation (2)) were calculated using the following formula:

(1) $$Diesel\;sorption\;capacity\;\left(g/g\right)=\frac{M_{a}-M_{b}}{M_{b}}$$

where *M_a_* is the mass of sample after sorption of diesel, and *M_b_* is the mass of sample before sorption [30].

(2) $$Efficiency\;of\;diesel/seawater\;removal\;\left(\%\right)=\frac{D_{f}-D_{i}}{D_{i}}\times 100\%$$

where *D_f_* is the final volume (mL) of diesel after sorption, and *D_i_* is the initial volume (mL) of diesel before sorption.

### 2.3. Screening

The samples were separated into three parts; stalk, spikelet, and whole and were rinsed with distilled water until all the debris was removed. After the cleaning procedure, the samples were sun-dried until they reached a constant weight. The samples were heated in a laboratory oven for 20 min at temperatures of 170 °C. After completion of the treatment, the heat was stopped, the samples were allowed to cool down at room temperature. The same method as in Section 2.2 was conducted and all the experiments were performed in triplicates. The selection of the part of EFB to be used in the subsequent parts of the study was based on the high efficiency of oil and low efficiency of water absorbed. From the result, the optimum part of EFB to absorb oil was analysed for characterization and morphological analysis.

### 2.4. Chemical Content Analysis and Sorbent Characterization

#### 2.4.1. Fourier Transform Infrared Spectroscopy (FTIR) Analysis

FTIR (ALPHA, Bruker Optik GmbH, Ettlingen, Germany) was used to determine the presence of functional groups in samples composition differences of untreated and treated samples before and after the filtration system [31].

#### 2.4.2. Morphology Analysis—Scanning Electron Microscope (SEM)

Observations on the surface morphological changes were carried out using SEM and Energy Dispersive X-ray (EDX) microanalysis to identify the composition of elements. The changes were observed by comparing the morphology of untreated and treated samples with the presence of diesel [31].

### 2.5. Statistical Experimental Design

#### 2.5.1. One-Factor-at-a-Time (OFAT)

Evaluation of optimum effects on efficiency of diesel–seawater sorption were carried out using the conventional OFAT approach based on four selected parameters: temperature (140, 150, 160, 170, 180, 190 °C), time (10, 15, 20, 25, 30, 35 min), packing density (0.06, 0.07, 0.08, 0.09, 0.10, 0.11 g/cm^3^), and oil concentration (5, 10, 15, 20, 25, 30% (*v*/*v*)). Each experiment was conducted in triplicates. The significant factors were analysed using one-way variance analysis (ANOVA) by GraphPad Prism 8.0.2 software (GraphPad Inc., San Diego, CA, USA). The influence of each parameter was tested using One-way ANOVA where significant and by pairwise post hoc comparisons using Tukey’s test [32].

#### 2.5.2. Response Surface Methodology (RSM)

Response Surface Methodology is a set of statistical and mathematical tools for designing experiments that minimise experimental runs. This study applied two experimental designs, Placket-Burman design (PBD) and Central Composite Design (CCD). By using analysis of variance (ANOVA), the adequacy of the model terms was obtained. The Fisher’s F test and ANOVA were used to find the significance of each model term. *p*-Values not more than 0.05 indicate the model terms are accepted as significant. Adequate precision greater than 4 is desirable to measure the signal-to-noise ratio and R^2^ values to determine the goodness of fit. Further studies on the efficiency of oil and water absorbed were carried out using the statistical approach of RSM [32].

##### Plackett–Burman Design

Four factors were picked from OFAT and analysed through the Plackett–Burman design. Each factor was tested at high (+1) and low (−1) levels (Table 1). The result shows 18 experimental runs with six centre points. The experimental design was developed and analysed using Design Expert 13.0.5.0 software (Stat-Ease Inc., Minneapolis, MN, USA) [32]. Equation (3) shows the PB factorial design at two levels:

(3) $$y=\beta_{0}+\overset{k}{\underset{i=1}{\sum }}\beta_{i}X_{i}$$

where *y* is the efficiency of diesel and seawater absorbed, *β*_0_ is the intercepted model and *β_i_* is the coefficient of linearity, *Χ_i_* is the independent variable’s coded level, and *k* is the number of variables.

##### Central Composite Design (CCD)

The CCD was applied to construct the response surface of the identified significant parameters with *p* values less than 0.05 [32]. As shown in Table 2, four significant variables were analysed with the combination of two factorial points (−1, +1), two axial points (−2, +2), and a sole central point (0). To predict the optimal conditions, the experimental response was fitted to a second-order polynomial regression model. Equation (4) quadratic mathematical model was used:

(4) $$y=\beta_{0}+\overset{k}{\underset{i=1}{\sum }}\beta_{i}x_{i}+\overset{k}{\underset{i=1}{\sum }}\beta_{ii}x_{i}^{2}+\overset{k}{\underset{1=i<j}{\sum }}\beta_{ij}x_{i}x_{j}$$

where *y* is the response variable, *x* is the independent factors that influence *y*, *β*_0_ is the intercept, *β_i_* is the *i*th linear coefficient, *β_ii_* is the quadratic coefficient, *β_ij_* is the coefficient of interaction effect, and *k* is the number of involved factors. Therefore, 30 experiment runs resulting from four significant variables with 6 centre points obtained were conducted. All experiments were done in triplicates.

## 3. Results and Discussion

### 3.1. Screening

**Figure 1:**
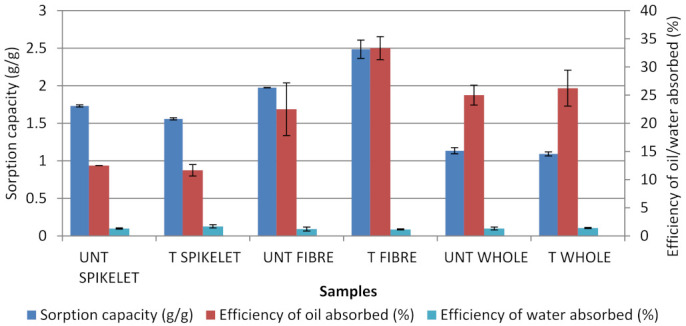
Screening part of EFB samples and efficiency of oil and water absorbed. UNT: Untreated; T: Treated.

Heat treatment at 170 °C in 20 min was used for all samples to find out the best optimum part with high efficiency of oil absorbed and low efficiency of water absorbed. Screening tests were conducted in mixed systems (water and oil mixture). Among the samples tested, Figure 1 shows that treated fibre (stalk) has the highest efficiency of oil absorbed at 33% (*p* < 0.0001) and the lowest efficiency of water absorbed at 1% (*p* < 0.0001). As treated fibre showed high efficiency of oil absorbed with low efficiency of water absorbed, it was selected for further study. 

For untreated and treated spikelets, for sorption capacity, and efficiency of oil and water absorbed, there were significant differences obtained: (F_6,14_ = 21.95, *p* < 0.0001), (F_6,14_ = 4.358, *p* = 0.0109) and (F_6,14_ = 3.830, *p* = 0.0180), respectively. Between untreated and treated EFB fibres, ANOVA analysis indicated the significant differences observed for the sorption capacity (F_6,14_ = 13.89, *p* < 0.0001). The efficiency of absorbed diesel (F_6,14_ = 2.842, *p* = 0.0503) and seawater (F_6,14_ = 4.842, *p* = 0.0071) between the EFB sorbent materials were found to have no significant differences. For untreated and treated whole EFB, the ANOVA analysis found that there were no significant difference for efficiency of oil and water absorbed: (F_6,14_ = 3.053, *p* = 0.0400) and (F_6,14_ = 0.5792, *p* = 0.7412), respectively, but there were significant differences obtained for sorption capacity (F_6,14_ = 38.02, *p* < 0.0001).

The evidence on the efficiency of the stalk to absorb more oil was due to the size of the natural fibres from the stalk that are larger in diameter, but have lower strength than spikelet [33]. Previous studies found that the galacturonic acid content in spikelets is lower than in stalk, which correlates with the lower cation content [33]. This fact, therefore, resulted in a difference in water absorptivity level between spikelet and stalk. Therefore, thermal treatment for spikelets was not expected to enhance the surface characteristic and sorption capacity. 

### 3.2. Chemical Content Analysis and Sorbent Characterisation

#### 3.2.1. Fourier Transform Infrared (FTIR) Spectroscopy Analysis

**Figure 2:**
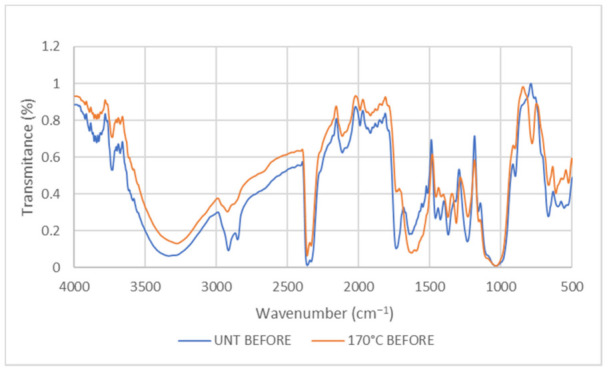
Comparison of IR spectra between EFB untreated and treated with 170 °C samples before wetting with oil. UNT: Untreated.

FTIR spectra for EFB untreated and treated at 170 °C before wetting with oil are presented in Figure 2. FTIR spectra illustrated the EFB fibres that were heat-treated at 170 °C, showing the evidence amounts of OH group stretching that shows a broad peak at 3279.63 cm^−1^, which contributes to the reduction of cellulose [34]. Stretching vibration of fingerprint at 2916.57 cm^−1^ illustrated the presence of C-H alkyl groups in the cellulose backbone [35]. With the heat-treated EFB, the vibration peaks at 2362.45 cm^−1^ weakened, thus concluding that the amounts of hydroxyl, hemicellulose, and cellulose were reduced when the EFB were exposed to the heat treatment [36]. The C=C stretching of the aromatic ring of lignin in untreated EFB at 1425.13 cm^−1^ was reduced to 1372.36 cm^−1^ after heat treatment, indicating the loss of lignin [37]. Thus, removing lignin, hemicellulose, and cellulose from heated samples led to the decreasing peak intensity in the spectra. The peak at the range of 1235.84 cm^−1^–665.04 cm^−1^ in untreated samples is higher than in treated samples at 1231.33 cm^−1^–662.84 cm^−1^, indicating C-O deformation. 

**Figure 3:**
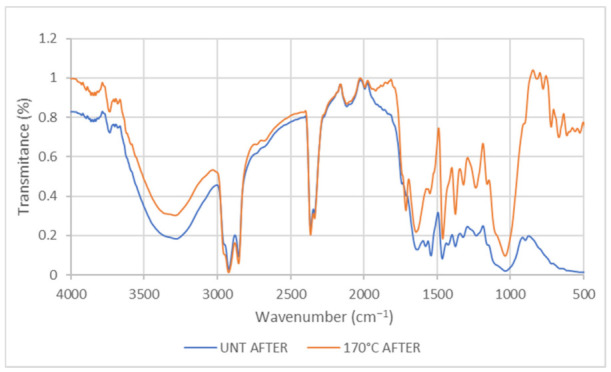
Comparison of IR spectra between EFB untreated and treated with 170 °C samples after wetting with oil. UNT: Untreated.

FTIR spectra for EFB untreated and treated at 170 °C after wetting with oil are presented in Figure 3. FTIR spectra showed the enhanced stretching band of the alkyl group after wetting with oil, indicating the presence of a long chain of alkyl from the hydrocarbon of diesel. The absence of ester linkage of carboxyl group in untreated and wetted with oil and the presence of ester linkage of carboxyl group in treated wetted with oil sample (1711.10 cm^−1^) indicates more sorption of oil in treated samples. These are the evidence of a hydrophobic functional group that confirms the sorption of diesel oil at the hydrophobic sites of the EFB [38,39]. Thus, it is confirmed that treated EFB fibre can undergo more sorption of diesel oil compared to untreated samples.

#### 3.2.2. Morphology Analysis-Scanning Electron Microscope (SEM)

**Figure 4:**
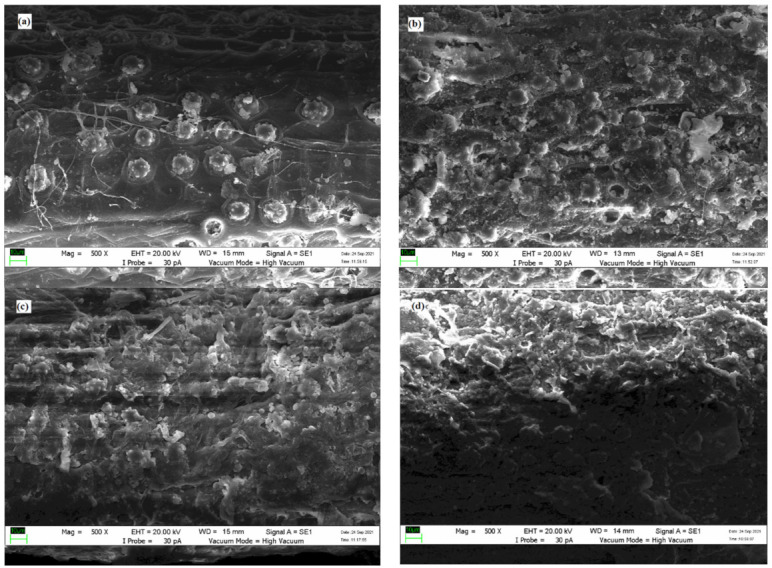
SEM images of samples (**a**) untreated, (**b**) treated with 170 °C at 20 min, (**c**) untreated wetted with oil, and (**d**) treated with 170 °C at 20 min wetted with oil at different magnification (500×).

Figure 4 illustrates SEM images of untreated, treated (170 °C, 20 min), untreated wetted with oil and treated (170 °C, 20 min) wetted with oil. Referring to Figure 4a of untreated samples, it was found that there were high amounts of ball-like structure on the surface of fibre attached to circular craters, which were described as silica in previous studies [31]. According to the earlier study, the compound of EFB fibre is also rich in inorganic elements such as silica consisting of silicon and oxygen [40,41]. Silica bodies can usually be found on the surface of woody plants [42]. Therefore, it is noted that a native EFB fibre has much silica on its surface.

For the heat-treated sample in Figure 4b, the SEM image spotted the removal of silica bodies on the EFB cell wall. According to Chin et al. [43], thermal modification contributes to lignin solubilization, resulting in silica bodies’ removal. This result is consistent with a previous study by Mohammad et al. [44], claiming that heat treatment of oil palm fibre at 170 °C led to the alteration of EFB surface and removal of silica bodies.

Figure 4c illustrates that the surface of EFB fibre was partially covered with oil. It was observed that the surface of untreated EFB fibre wetted with oil was rough. Meanwhile, Figure 4d illustrates that the pores from the removal of silica bodies were covered completely. The surface of treated EFB fibre wetted with oil was found smoother than untreated wetted with oil sample. Therefore, it was believed that diesel was successfully sorbed on the surface of treated EFB fibre.

### 3.3. Optimisation of Oil Absorbed Using One-Factor-at-a-Time (OFAT)

#### 3.3.1. Effects of Temperature and Time

**Figure 5:**
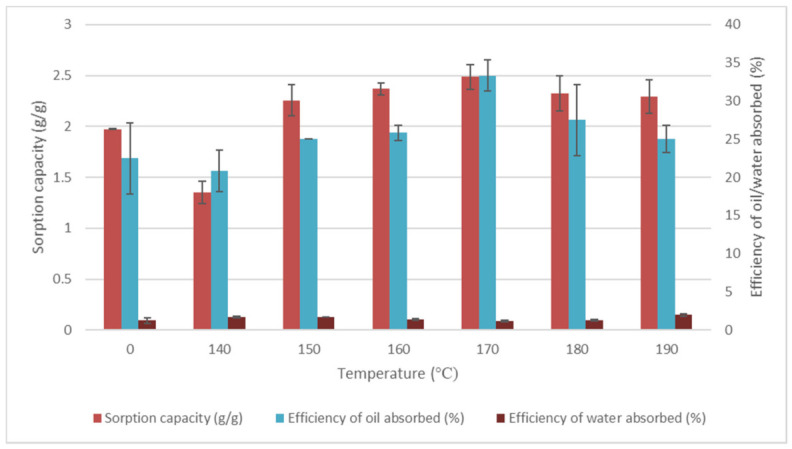
Effects of temperature on EFB treated fibre. Data obtained were the average efficiency of oil absorbed (%), the efficiency of water absorbed (%), and sorption capacity (g/g) on temperature. Vertical bars indicate SEM of three replicates.

**Figure 6:**
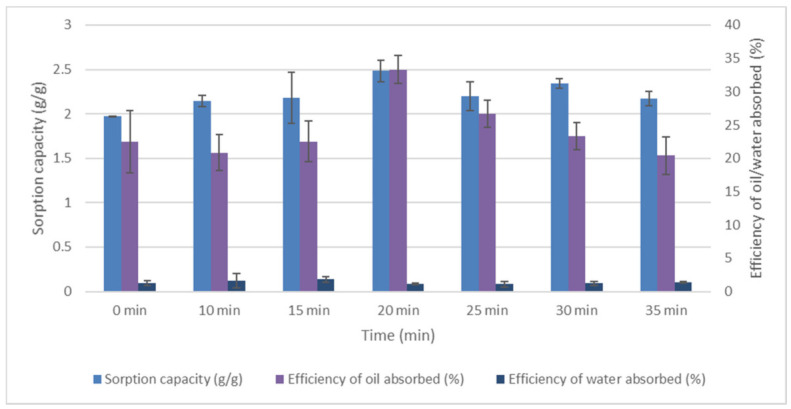
Effects of time on EFB treated fibre. Data obtained were the average efficiency of oil absorbed (%), the efficiency of water absorbed (%), and sorption capacity (g/g) on temperature. Vertical bars indicate SEM of three replicates.

Temperature and time significantly influenced the efficiency of oil absorbed, water absorbed, and sorption capacity. It was observed in Figure 5 that treated EFB fibre has higher oil absorption than untreated EFB sorbents. In treated samples, the temperatures of 140 °C, 150 °C, 160 °C, 170 °C, 180 °C, and 190 °C led to a significant amount of oil absorbed and lower amount of water absorbed. Figure 6 illustrates the effects of heating time on EFB treated fibre. In different heating times, the results showed that the efficiency of oil absorbed was the highest at 20 min. Thus, the 170 °C and 20 min heating time recorded the highest efficiency of oil absorbed and the lowest efficiency of water absorbed. 

Fibre surface modification using thermal treatment was done to strengthen the poor properties of the surface by reducing the polar components, which can attain good adhesion in the fibre matrix and improve the interface of the fibre matrix [45]. Thus, it was proven that the efficiency in absorbing oil increased by up to 170 °C with heat-treated fibres. Sreekumar et al. [46] suggested that fibre surface modification by using thermal treatment successfully decreased the water absorption, supporting a more significant fibre interaction. At a low temperature and time of heating, the hydrophobic properties of the sorbent are not affected. The adsorption of oil at low quantities using low-temperature sorbents is caused by removing surface impurities that ease the oil adsorption process [47]. On the other hand, thermal treatment of sorbent at high temperatures results in sorbent carbonisation, thus enhancing the oil sorption capacity [48]. Similar findings were reported by Sreekumar et al. [46], Husseien et al. [49], Kudaybergenov et al. [50], and Kudaybergenov et al. [51] on the efficiency of using a thermally modified sample as biosorbent to remediate pollution.

#### 3.3.2. Effects of Packing Density

**Figure 7:**
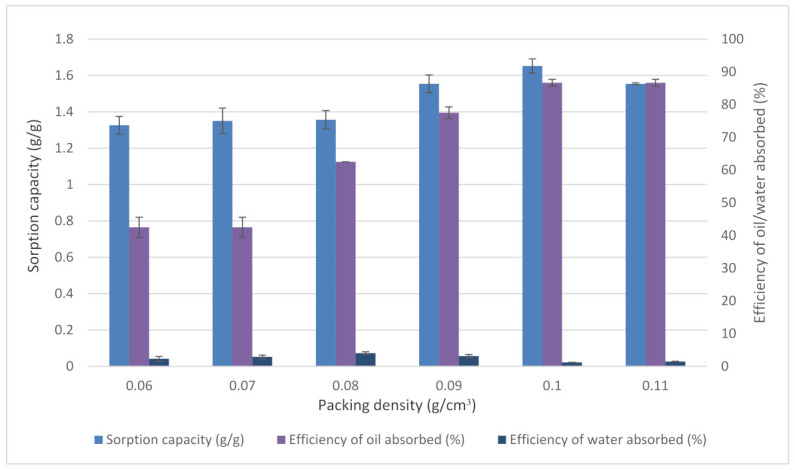
Effects of packing density on EFB treated fibre. Data obtained were the average efficiency of oil absorbed (%), the efficiency of water absorbed (%), and sorption capacity (g/g) on temperature. Vertical bars indicate SEM of three replicates.

Packing density significantly influenced the efficiency of oil or water absorbed and sorption capacity (Figure 7). The result indicates that the increase of packing density affected the efficiency of oil absorbed up to 0.1 g/cm^3^. A further increase to 0.11 g/cm^3^ did not increase the oil absorbed and sorption capacity. The decrease of oil absorbed after a packing density of 0.1 g/cm^3^ was due to less space available for oil sorption [52]. At low packing density, the efficiency of oil absorbed is low. This was made evident from the size of pores between the inter-fibre distance that became large due to non-compacted samples, causing the fibre not to have sufficient capillary pressure to absorb oil [53]. At the loose packing condition such as 0.06 g/cm^3^ and 0.07 g/cm^3^, there was an insufficient utilisation of samples that resulted in low oil absorption [54]. It was found that higher and lower packing density corresponded to lower oil absorbed and sorption capacity. 

#### 3.3.3. Effects of Oil Concentration

**Figure 8:**
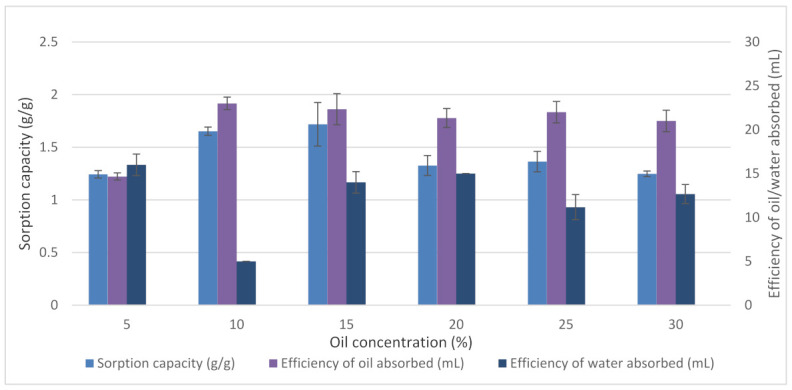
Effects of oil concentration on EFB treated fibre. Data obtained were the average efficiency of oil absorbed (%), the efficiency of water absorbed (%), and sorption capacity (g/g) on temperature. Vertical bars indicate SEM of three replicates.

The initial oil concentration significantly influenced the efficiency of oil and water absorbed. Figure 8 indicates that increasing the initial diesel concentration significantly affected the absolute amount of diesel absorbed up to a concentration of 10%. The result showed that oils concentration above 10% did not lead to any further increase in diesel absorption. 

At a low diesel concentration, the increase in diesel absorbed is claimed due to the adsorption of diesel molecules at the hydrophobic reactive sites [55]. Moreover, oil molecules are also believed to adsorb into the hollow lumen of the EFB sorbents [55]. According to Kudaybergenov et al. [50], when the sorbents are exposed to oil, the oil will be absorbed by the macropores and micropores of the sorbents until equilibrium is reached. When equilibrium is reached due to the saturation of oil molecules at reactive sites of sorbents, desorption occurs [56]. It was found that the oil concentration corresponded to oil absorbed until it reached an equilibrium state.

### 3.4. Optimisation of Oil Absorbed Using Response Surface Methodology (RSM)

#### 3.4.1. Plackett–Burman Design

Eighteen runs were generated from the PB design (Table 3). In run 2, the highest value of oil-absorbed efficiency was obtained at the condition of 190 °C, 15 min, 0.11 g/cm^3^ with 15% diesel and the lowest value of oil-absorbed efficiency was 160 °C, 30 min, 0.11 g/cm^3^ with 5% diesel.

From the ANOVA, the analysis identified that temperature, time, packing density, and oil concentration significantly influenced diesel sorption. Table 4 shows the ANOVA table for the oil absorbed from PBD. The results suggest that the overall model was highly significant, with an F value of 33.37 and a *p*-value less than 0.05.

#### 3.4.2. Central Composite Design (CCD)

CCD results of 30 runs are listed in Table 5. The highest oil absorbed was obtained at the condition of 175 °C, 22.5 min, and 0.095 g/cm^3^ with 10% diesel. The lowest oil absorbed was obtained at 175 °C, 22.5 min, and 0.095 g/cm^3^ with 0% diesel.

In CCD, ANOVA was conducted to detect the significance of each model term. The quadratic model was applied in this design. The analysis shown in Table 6 confirmed that the model is significant (*p* < 0.0001).

Based on Table 6, the pairs of variables showed no interaction, thus concluding that all parameters are independent. Supposing the interaction between variables is presented from the model graph, a clear peak appears in the centre of a response surface plot and is suggested as a mutual relationship between the two variables [57]. Packing density and oil concentration were vital factors influencing the absorption of diesel oil in EFB. The data obtained here clearly show that oil concentration achieved a more significant result than temperature, time, and packing density. This is consistent with the studies of Thompson et al. [56] and Onwuka et al. [31], mentioning that sorbent’s efficiency in absorbing oil depends on the initial oil concentration. Packing density also gave a significant result consistent with a previous study that concluded the enhanced availability of the sorbent to absorb oil with the increase of samples’ packing density [54]. 

**Figure 9:**
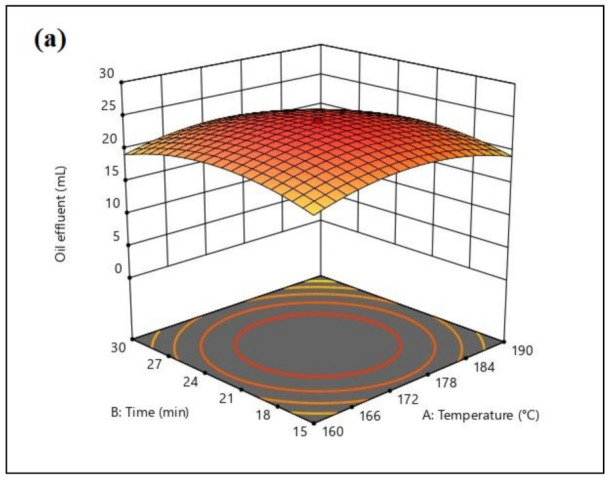

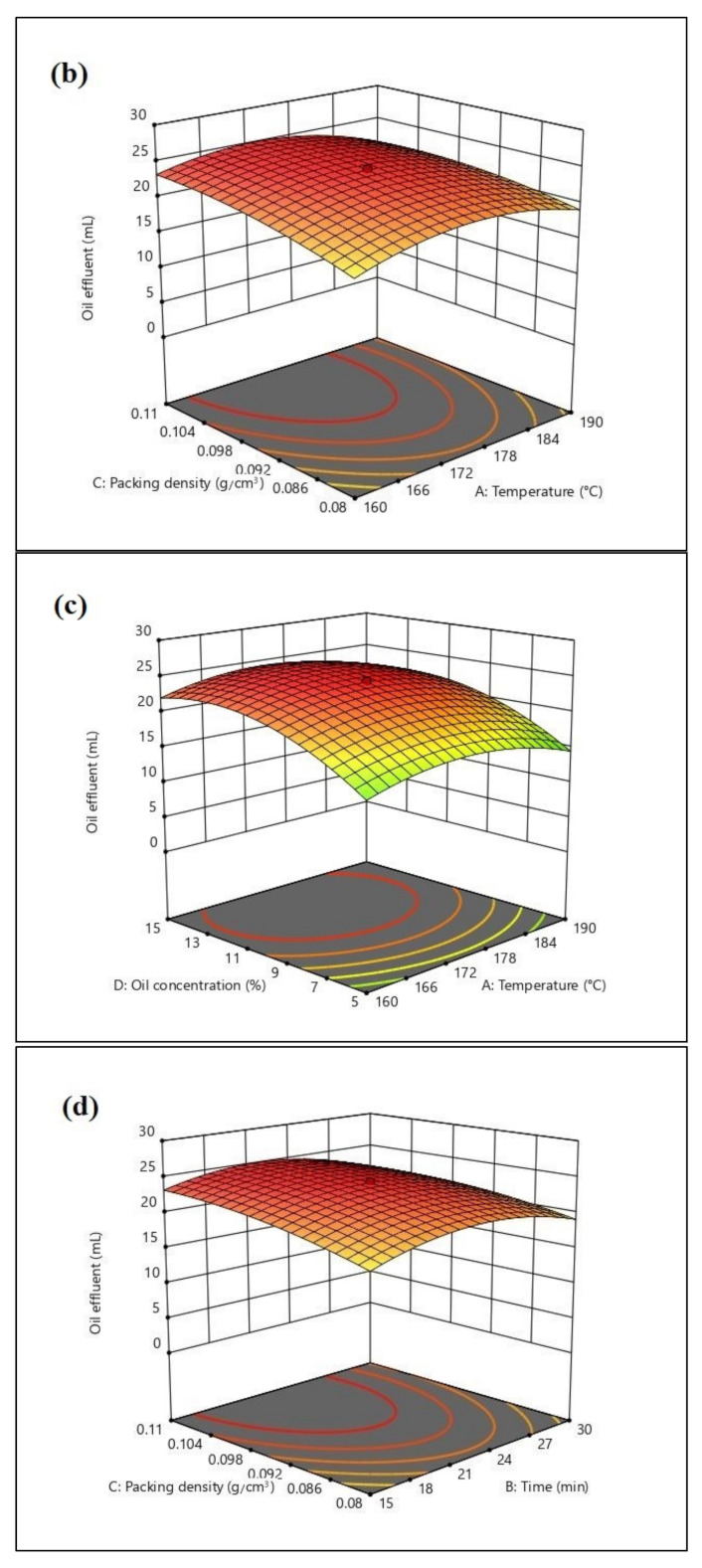

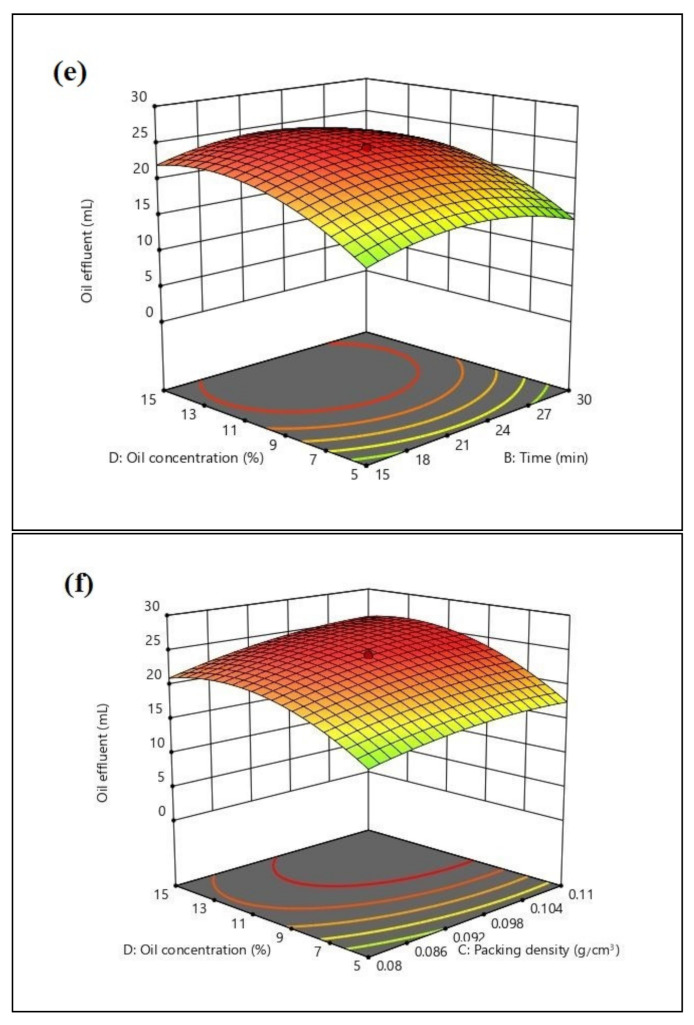
3D Contour plots generated by Design Expert (Stat Ease, Inc., Minneapolis, USA) of the significantly interacting model terms: (**a**) A: temperature and B: time, (**b**) C: packing density and A: temperature, (**c**) D: oil concentration and A: temperature, (**d**) C: packing density and B: time, (**e**) D: oil concentration and B: time, and (**f**) D: oil concentration and C: packing density.

Three-dimensional response surface plots were constructed by plotting the oil absorbed as a response on the z-axis against two independent parameters. The model graph of temperature and time generated by CCD is given in Figure 9a. The combination of optimum temperature (170–175 °C) and time (20–23 min) led to the highest oil absorption, reaching 24 mL. At high and low temperatures, as well as the time of heating, oil absorbed was lower. Figure 9b shows the model graph of packing density and temperature. The result indicates that the increase of packing density affected the efficiency of oil absorbed up to 0.1 g/cm^3^. A further increase to 0.11 g/cm^3^ did not increase the oil absorbed as the graph remained constant. Figure 9c illustrates the model graph of oil concentration and temperature in which the higher oil absorption was observed at a temperature of 166–178 °C and diesel concentration of 11–13%. In the packing density and time model graph, the higher oil absorption was obtained at a heating time of 21–24 min with a packing density of 0.104 g/cm^3^ (Figure 9d). Figure 9e shows at a heating time of 21–24 min, the oil concentration in the range of 11–13% diesel results in higher oil absorption. In Figure 9f, the model graph between packing density and oil concentration is illustrated. The higher oil absorption of 25.984 mL was observed at a packing density of 0.098–0.104 g/cm^3^ and 11–13% diesel.

The model was validated by conducting the experimental trial by referring to the predicted conventional OFAT and statistical RSM (Table 7). The predicted value of OFAT was 23 mL of oil absorbed and 5 mL of water absorbed, whereas the predicted value of RSM was 24 mL of oil absorbed and 8.178 mL of water absorbed. The experimental condition suggested by the software was applied and yielded 24.33 mL of oil absorbed and 8.333 mL of water absorbed. The data indicated that RSM provides a better approach to obtain a high efficiency of oil absorbed.

## 4. Conclusions

This study is the first to apply a statistical experimental design to optimise oil absorbed using EFB stalk fibre, sampled from Manjung, Malaysia. The optimisation of the efficiency of oil absorbed through conventional OFAT and the statistical approach of RSM resulted in 23 mL and 25.33 mL of oil absorbed, respectively. In conclusion, the results obtained support the statistical RSM for being an effective tool to optimise the factors to improve oil absorbed compared to the conventional OFAT approach. Further in-depth studies of EFB as sorbent material for oil pollution will further enhance the application of agricultural waste as a tool for bioremediation.

## Figures and Tables

**Table 1:** Experimental values and levels of variables tested for EFB fibre as biosorbent in Plackett–Burman design.

Variables	Code	Unit	Experimental Range	
			Low (−1)	High (+1)
Temperature	A	°C	160	190
Time	B	min	15	30
Packing density	C	g/cm^3^	0.08	0.11
Oil concentration	D	% (*v*/*v*)	5	15

**Table 2:** Experimental values and levels of the selected independent factors for CCD optimisation.

	Symbol	Unit	Experimental Values				
			−2	−1	0	+1	+2
Temperature	A	°C	145	160	175	190	205
Time	B	min	7.5	15	22.5	30	37.5
Packing density	C	g/cm^3^	0.065	0.08	0.095	0.11	0.125
Oil concentration	D	% (*v*/*v*)	0	5	10	15	20

Note: Factorial points (−1, +1); Axial points (−2, +2); Sole central point (0).

**Table 3:** Screening of significant parameters affecting oil and water effluent using Plackett– Burman.

Run	A	B	C	D	Oil Absorbed	Water Absorbed
1	190	15	0.08	5	14.6667	16.6667
2	190	15	0.11	15	25	10
3	175	22.5	0.095	10	24	11
4	160	30	0.11	5	9.33333	15.6667
5	175	22.5	0.095	10	24	11
6	175	22.5	0.095	10	24	11
7	160	30	0.08	15	14.667	7.3333
8	175	22.5	0.095	10	24	11
9	160	15	0.08	5	12	12
10	190	30	0.08	15	17.1667	10.3333
11	160	30	0,11	15	22.3333	10
12	160	15	0.11	5	13.3333	20
13	175	22.5	0.095	10	24	11
14	190	30	0.08	5	12.6667	11.6667
15	190	15	0.11	15	25	10
16	160	15	0.08	15	19.6667	11
17	190	30	0.11	5	15	16.3333
18	175	22.5	0.095	10	24	11

A: Temperature (°C); B: Time (min); C: Packing density (g/cm^3^); D: Oil concentration (%).

**Table 4:** ANOVA for the oil absorbed of the PBD model used to identify the factor significantly influencing diesel sorption.

Source	Sum of Squares	DF	Mean Square	F Value	*p* Value
Model	269.42	4	67.35	33.37	˂0.0001
A	27.50	1	27.50	13.63	0.0031
B	28.52	1	28.52	14.13	0.0027
C	30.61	1	30.61	15.17	0.0021
D	182.78	1	182.78	90.56	˂0.0001
Residual	24.22	12	2.02	104.57	
Cor Total	504.69	17			
Std. Dev.	1.42		R^2^	0.9175	
Mean	19.16		Adjusted R^2^	0.8900	
C.V.	7.42		Predicted R^2^	0.7576	
			Adequate Precision	17.1700	

**Table 5:** Optimisation of parameters for diesel sorption by EFB fibre using central composite design (CCD).

Run Order	A	B	C	D	Oil Absorbed (mL)	
					Experimental Value	Predicted Value
1	175	22.5	0.095	10	24.30	24.20
2	190	15	0.11	5	13.00	12.56
3	175	22.5	0.065	10	14.00	16.51
4	175	22.5	0.095	10	24.30	24.20
5	175	22.5	0.095	10	24.00	24.20
6	160	30	0.08	15	17.33	15.98
7	190	30	0.08	5	11.33	10.31
8	190	30	0.11	15	19.67	18.48
9	175	22.5	0.095	0	0.000	3.569
10	190	15	0.08	15	16.67	16.01
11	175	7.5	0.095	10	15.00	14.35
12	160	30	0.11	15	20.00	20.76
13	175	22.5	0.095	10	24.00	24.20
14	175	22.5	0.095	20	18.00	17.68
15	160	15	0.11	15	22.67	21.90
16	190	15	0.11	15	19.67	20.45
17	175	37.5	0.095	10	9.500	13.40
18	160	15	0.08	5	9.333	8.729
19	205	22.5	0.095	10	10.00	13.18
20	175	22.5	0.095	10	24.27	24.20
21	160	15	0.11	5	13.33	13.67
22	145	22.5	0.095	10	14.00	14.07
23	190	15	0.08	5	12.33	10.12
24	175	22.5	0.125	10	23.00	23.74
25	190	30	0.08	15	18.00	16.20
26	160	30	0.11	5	13.67	12.53
27	160	30	0.08	5	12.00	9.757
28	160	15	0.08	15	13.33	14.95
29	175	22.5	0.095	10	24.33	24.20
30	190	30	0.11	5	13.67	10.59

A: Temperature (°C); B: Time (min); C: Packing density (g/cm^3^); D: Oil concentration (%).

**Table 6:** Results of ANOVA for CCD model identifying factors and pairwise interactions significantly influencing diesel absorption.

Source	Sum of Squares	df	Mean Square	F-value	*p* Value	
Model	936.57	14	66.90	12.65	<0.0001	significant
A	1.19	1	1.19	0.2241	0.6427	
B	1.34	1	1.34	0.2530	0.6223	
C	78.24	1	78.24	14.80	0.0016	
D	298.69	1	298.69	56.48	<0.0001	
AB	0.6945	1	0.6945	0.1313	0.7221	
AC	6.25	1	6.25	1.18	0.2941	
AD	0.1111	1	0.1111	0.0210	0.8867	
BC	4.69	1	4.69	0.8878	0.3610	
BD	1.806 × 10^−9^	1	1.806 × 10^−9^	3.416 × 10^−10^	1.0000	
CD	4.00	1	4.00	0.7576	0.3981	
A^2^	191.69	1	191.69	36.25	<0.0001	
B^2^	182.73	1	182.73	34.56	<0.0001	
C^2^	28.46	1	28.46	5.38	0.0349	
D^2^	315.88	1	315.88	59.74	<0.0001	
Residual	79.32	15	5.29			
Lack of Fit	79.20	10	7.92	326.32	<0.0001	Significant
Pure Error	0.1213	5	0.0243			
Cor Total	1015.89	29				
			R^2^		0.9219	
Std. Dev.	2.30		Adjusted R^2^		0.8491	
Mean	16.49		Predicted R^2^		0.5508	
C.V.%	13.95		Adequate Precision		12.6874	

**Table 7:** Validation model using the predicted optimum value in OFAT and RSM.

Optimised Parameters	Predicted OFAT	Predicted RSM	Experimental RSM
Temperature (°C)	170	175	175
Time (min)	20	22.5	22.5
Packing density (g/cm^3^)	0.1	0.095	0.095
Oil concentration (%)	10%	10	10
Oil absorbed (mL)	23	24	24.33
*p* value	<0.0001 (significant)	<0.0001 (significant)	<0.0001 (significant)
Water absorbed (mL)	5	8.178	8.333
*p* value	<0.0001 (significant)	0.0012 (significant)	0.0398 (significant)

## References

[1] Errington I., King C.K., Wilkins D., Spedding T., Hose G.C. (2018). Ecosystem effects and the management of petroleum-contaminated soils on sub-Antarctic islands. Chemosphere.

[2] Neuman S. Oil Spill Reported Off China Coast after Tanker, Bulk Carrier Collide 2021. https://www.npr.org/2021/04/27/991144729/oil-spill-reported-off-china-coast-after-tanker-bulk-carrier-collide.

[3] Macoustra G.K., King C.K., Wasley J., Robinson S.A., Jolley D.F. (2015). Impact of hydrocarbons from a diesel fuel on the germination and early growth of subantarctic plants. Environ. Sci. Process Impacts.

[4] D’Andrea M.A., Reddy G.K. (2014). Crude Oil Spill Exposure and Human Health Risks. J. Occup. Environ. Med..

[5] Troisi G.M., Bexton S., Robinson I. (2006). Polyaromatic Hydrocarbon and PAH metabolite burdens in oiled common guillemots (Uria aalge) Stranded on the East Coast of England (2001–2002). Environ. Sci. Technol..

[6] Pohjola S.K., Savela K., Kuusimäki L., Kanno T., Kawanishi M., Weyand E. (2004). Polycyclic aromatic hydrocarbons of diesel and gasoline exhaust and DNA adduct detection in calf thymus DNA and lymphocyte DNA of workers exposed to diesel exhaust. Polycycl. Aromat. Compd..

[7] Khanna S., Gharpure A.S. (2017). Petroleum carcinogenicity and aerodigestive tract: In context of developing nations. Cureus.

[8] Li H., Boufadel M.C. (2010). Long-term persistence of oil from the Exxon Valdez spill in two-layer beaches. Nat. Geosci..

[9] Afenyo M., Veitch B., Khan F. (2016). A state-of-the-art review of fate and transport of oil spills in open and ice-covered water. Ocean Eng..

[10] Li X., Li J., Qu C., Yu T., Du M. (2021). Bioremediation of clay with high oil content and biological response after restoration. Sci. Rep..

[11] Trejos-Delgado C., Cadavid-Restrepo G., Hormaza-Anaguano A., Agudelo E., Barrios-Ziolo L., Loaiza-Usuga J.C., Cardona-Gallo S.A. (2020). Oil bioremediation in a tropical contaminated soil using a reactor. An. Acad. Bras. Ciênc..

[12] Yousefi K., Mohebbi A., Pichtel J. (2021). Biodegradation of weathered petroleum hydrocarbons using organic waste amendments. Appl. Environ. Soil Sci..

[13] Anastopoulos I., Bhatnagar A., Lima E.C. (2016). Adsorption of rare earth metals: A review of recent literature. J. Mol. Liq..

[14] Geng N., Wu Y., Zhang M., Tsang D.C., Rinklebe J., Xia Y., Ok Y.S. (2019). Bioaccumulation of potentially toxic elements by submerged plants and biofilms: A Critical review. Environ. Int..

[15] Zamparas M., Tzivras D., Dracopoulos V., Ioannides T. (2020). Application of Sorbents for Oil Spill Cleanup Focusing on Natural-Based Modified Materials: A Review. Molecules.

[16] Abdullah N., Sulaim F. (2013). The Oil Palm Wastes in Malaysia. Biomass Now-Sustainable Growth and Use.

[17] Husseien M., Amer A.A., El-Maghraby A., Hamedallah N. (2009). A comprehensive characterization of corn stalk and study of carbonized corn stalk in dye and gas oil sorption. J. Anal. Appl. Pyrolysis.

[18] Cao S., Dong T., Xu G., Wang F. (2017). Oil Spill Cleanup by Hydrophobic Natural Fibers. J. Nat. Fibers.

[19] Abdelwahab O., Nasr S.M., Thabet W.M. (2017). Palm fibers and modified palm fibers adsorbents for different oils. Alex. Eng. J..

[20] Cheu S.C., Kong H., Song S.T., Saman N., Johari K., Mat H. (2016). High removal performance of dissolved oil from aqueous solution by sorption using fatty acid esterified pineapple leaves as novel sorbents. RSC Adv..

[21] Mba O.I., Dumont M., Ngadi M. (2015). Palm oil:Processing, characterization and utilization in the food industry—A review. Food Biosci..

[22] Alam F.A.S.A., Begum H. (2015). Malaysian oil palm industry: Prospect and problem. J. Food Agric. Environ..

[23] Chavalparit O., Rulkens W.H., Mol A.P.J., Khaodhair S. (2006). Options for environmental sustainability of the crude palm oil industry in Thailand through enhancement of industrial ecosystems. Environ. Dev. Sustain..

[24] Jahi N., Othaman R., Mat Lazim A., Ramli S. (2020). Empty fruit Bunch Cellulose Based Sorbent for OIL Sorption in palm oil Mill Effluent. Sains Malays..

[25] Dungani R., Aditiawati P., Aprilia S., Yuniarti K., Karliati T., Suwandhi I., Sumardi I. (2018). Biomaterial from Oil Palm Waste: Properties, Characterization and Applications. Palm Oil.

[26] Siti Aisyah M.S., Uemura Y., Yusup S. (2014). The Effect of Alkaline Addition in Hydrothermal Pretreatment of Empty Fruit Bunches on Enzymatic Hydrolysis Efficiencies. Procedia Chem..

[27] Astimar A.A., Husin M., Anis M. (2002). Preparation of cellulose from oil palm empty fruit bunches via ethanol digestion: Effect of acid and alkali catalyst. J. Oil Palm Res..

[28] Daneshfozoun S., Abdullah M.A., Abdullah B. (2017). Preparation and characterization of magnetic biosorbent based on oil palm empty fruit bunch fibers, cellulose and Ceiba pentandra for heavy metal ions removal. Ind. Crops Prod..

[29] Joseph C.G., Daud W.M.A.W., Shane Q.K., Sanmugam K. (2015). Parametric and adsorption kinetic studies of reactive black 5 removal from textile simulated wastewater using oil palm (*Elais guineensis*) empty fruit Bunch. J. Appl. Sci..

[30] Ibrahim S., Baharuddin S.N.I.B., Ariffin B., Hanafiah M.A.K.M., Kantasamy N. (2018). Cogon grass for oil sorption: Characterization and sorption studies. Key Eng. Mater..

[31] Onwuka J.C., Agbaji E.B., Ajibola V.O., Okibe F.G. (2018). Treatment of crude oil-contaminated water with chemically modified natural fiber. Appl. Water Sci..

[32] Zakaria N.N., Gomez-Fuentes C., Abdul Khalil K., Convey P., Roslee A.F.A., Zulkharnain A., Sabri S., Shaharuddin N.A., Cardenas L., Ahmad S.A. (2021). Statistical optimisation of diesel biodegradation at low temperatures by an Antarctic marine bacterial consortium isolated from non-contaminated seawater. Microorganisms.

[33] Reneta Nafu Y., Foba-Tendo J., Njeugna E., Oliver G., Omar Cooke K. (2015). Extraction and characterization of fibres from the stalk and spikelets of empty Fruit Bunch. J. Appl. Chem..

[34] Khalil H.S., Ismail H., Rozman H.D., Ahmad M.N. (2001). The effect of acetylation on interfacial shear strength between plant fibres and various matrices. Eur. Polym. J..

[35] Xiao X., Bian J., Li M.F., Xu H., Xiao B., Sun R.C. (2014). Enhanced enzymatic hydrolysis of bamboo (*Dendrocalamus gigantus* munro) culm by hydrothermal pretreatment. Biores. Technol..

[36] Hao Y., Pan Y., Du R., Wang Y., Chen Z., Zhang X., Wang X. (2018). The influence of a thermal treatment on the decay resistance of wood via FTIR Analysis. Adv. Mater. Sci. Eng..

[37] Rosli N.S., Harun S., Jahim J.M., Othaman R. (2017). Chemical and physical characterization of oil palm empty fruit bunch. Malays. J. Anal. Sci..

[38] Ibrahim S., Ha-Ming A., Wang S. (2009). Removal of emulsifed food and mineral oils from wastewater using surfactant modifed barley straw. Biores. Technol..

[39] Isroi C.A., Panji T., Wibowo N.A., Syamsu K. (2017). Bioplastic production from cellulose of oil palm empty fruit bunch. IOP Conf. Ser. Earth Environ. Sci..

[40] Law K.N., Daud W.W., Ghazali A. (2007). Morphology and chemical nature of fiber strands of oil palm empty fruit bunch (OPEFB). Bioresources.

[41] Lins U., Barros C.F., Da Cunha M., Miguens F.C. (2002). Structure, morphology and composition of silicon biocomposites in the palm tree *Syagrus coronata* (Mart) Becc. Protoplasma.

[42] Yoon C.J., Kim K.W. (2008). Anatomical descriptions of silicified woods from Madagascar and Indonesia by scanning electron microscopy. Micron.

[43] Chin S.X., Chia C.H., Zakaria S., Fang Z., Ahmad S. (2015). Ball milling pretreatment and diluted acid hydrolysis of oil palm empty fruit bunch (EFB) fibres for the production of levulinic acid. J. Taiwan Inst. Chem. Eng..

[44] Mohammad I.N., Ongkudon C.M., Misson M. (2020). Physicochemical Properties and Lignin Degradation of Thermal-Pretreated Oil Palm Empty Fruit Bunch. Energies.

[45] Ahmad R., Hamid R., Osman S.A. (2019). Physical and chemical modifications of plant fibres for reinforcement in cementitious composites. Adv. Civ. Eng..

[46] Sreekumar P.A., Thomas S.P., Saiter J., Joseph K., Unnikrishnan G., Thomas S. (2009). Effect of fiber surface modification on the mechanical and water absorption characteristics of sisal/polyester composites fabricated by resin transfer molding. Compos. Part A Appl. Sci. Manuf..

[47] El Gheriany I.A., Ahmad El Saqa F., Abd El Razek Amer A., Hussein M. (2020). Oil spill sorption capacity of raw and thermally modified orange peel waste. Alex. Eng. J..

[48] Angelova D., Uzunov I., Uzunova S., Gigova A., Minchev L. (2011). Kinetics of oil and oil products adsorption by carbonized rice husks. Chem. Eng. J..

[49] Husseien M., Amer A.A., El-Maghraby A., Taha N.A. (2008). Experimental Investigation of Thermal Modification Influence on Sorption Qualities of Barley Straw. J. Appl. Sci. Res..

[50] Kudaybergenov K.K., Ongarbayev E.K., Mansurov Z.A. (2012). Thermally treated rice husks for petroleum adsorption. Int. J. Biol. Chem..

[51] Kudaybergenov K.K., Ongarbayev E.K., Mansurov Z.A. (2010). Carbonaceous composites from agricultural wastes for adsorption of hydrocarbon contamination in water. Eurasian Chem. J..

[52] Xu Y., Shen H., Xu G. (2019). Evaluation of oil sorption kinetics behavior and wetting characteristic of cattail fiber. Cellulose.

[53] Zhu L., Perwuelz A., Lewandowski M., Campagne C. (2008). Static and dynamic aspects of liquid capillary flow in thermally bonded polyester nonwoven fabrics. J. Adhes. Sci. Technol..

[54] Dong T., Wang F., Xu G. (2014). Theoretical and experimental study on the oil sorption behavior of kapok assemblies. Ind. Crops Prod..

[55] Wang J.T., Zheng Y.A., Wang A.Q. (2012). Effect of kapok fibre treated with various solvents on oil absorbency. Ind. Crops. Prod..

[56] Thompson N.E., Emmanuel G.C., Adagadzu K.J., Yusuf N.B. (2010). Sorption studies of crude oil on acetylated rice husks. Scholars research library. Arch. Appl. Sci. Res..

[57] Zhou J., Yu X., Ding C., Wang Z., Zhou Q., Pao H., Cai W. (2011). Optimization of phenol degradation by *Candida tropicalis* using Plackett-Burman design and response surface methodology. J. Environ. Sci..

